# The Light Sword Lens - A novel method of presbyopia compensation: Pilot clinical study

**DOI:** 10.1371/journal.pone.0211823

**Published:** 2019-02-04

**Authors:** Krzysztof Petelczyc, Anna Byszewska, Ewelina Chojnacka, Zbigniew Jaroszewicz, Karol Kakarenko, Alejandro Mira-Agudelo, Aleksandra Ostrowska-Spaleniak, Aleksandra Składowska, Andrzej Kołodziejczyk, Marek Rękas

**Affiliations:** 1 Faculty of Physics, Warsaw University of Technology, Warsaw, Poland; 2 Ophthalmology Department, Military Institute of Medicine, Warsaw, Poland; 3 Institute of Applied Optics, Warsaw, Poland; 4 National Institute of Telecommunications, Warsaw, Poland; 5 Facultad de Ciencias Exactas y Naturales, Universidad de Antioquia, Medellin, Colombia; 6 Department of Neurophysiology, Nencki Institute of Experimental Biology, Warsaw, Poland; University of Debrecen, Faculty of Medicine, HUNGARY

## Abstract

**Purpose:**

Clinical assessment of a new optical element for presbyopia correction–the Light Sword Lens.

**Methods:**

Healthy dominant eyes of 34 presbyopes were examined for visual performance in 3 trials: reference (with lens for distance correction); stenopeic (distance correction with a pinhole ϕ = 1.25 mm) and Light Sword Lens (distance correction with a Light Sword Lens). In each trial, visual acuity was assessed in 7 tasks for defocus from 0.2D to 3.0D while contrast sensitivity in 2 tasks for defocus 0.3D and 2.5D. The Early Treatment Diabetic Retinopathy Study protocol and Pelli-Robson method were applied. Within visual acuity and contrast sensitivity results degree of homogeneity through defocus was determined. Reference and stenopeic trials were compared to Light Sword Lens results. Friedman analysis of variance, Nemenyi post-hoc, Wilcoxon tests were used, p-value < 0.05 was considered significant.

**Results:**

In Light Sword Lens trial visual acuity was stable in tested defocus range [20/25–20/32], Stenopeic trial exhibited a limited range of degradation [20/25–20/40]. Light Sword Lens and reference trials contrast sensitivity was high [1.9–2.0 logCS] for both defocus cases, but low in stenopeic condition [1.5–1.7 logCS]. Between-trials comparisons of visual acuity results showed significant differences only for Light Sword Lens versus reference trials and in contrast sensitivity only for Light Sword Lens versus stenopeic trials.

**Conclusions:**

Visual acuity achieved with Light Sword Lens correction in presbyopic eye is comparable to stenopeic but exhibits none significant loss in contrast sensitivity. Such correction method seems to be very promising for novel contact lenses and intraocular lenses design.

## Introduction

Nowadays presbyopia is a genuine problem of the world's population. There are over 900 million people aged 60 or more all over the world, and this number is expected to rise one and half times within the next 15 years. [1] Prolongation of the working age in the context of aging of the human eye, requires compensation of accommodation mechanism by correction with artificial lenses (or systems) enabling good quality of vision at all functional distances. [2] There are various spectacles designs and contact (CL) or intraocular (IOL) lenses for compensation of presbyopia due to aging or as a consequence of cataract surgery. [3, 4] Multifocal CLs and IOLs with symmetry of revolution are the most widespread and frequently used e.g. CL: Acuvue Oasys for Presbyopia (Johnson & Johnson), Biofinity Multifocal (Cooper Vision), Dailies AquaComfort Plus (Alcon), IOL: Alcon Panoptix (Alcon), Tecnis Symfony (Abbott Medical Optics). Symmetry of revolution means that their shape is fully defined by one cross-section through the center [5, 6] due to placement of annular areas of disparate power in the center and periphery or some set of diffractive rings. Another approach consists of refractive elements with angularly varying optical power, termed Light Sword Lenses (LSLs), [7–9] which are subject of this study. Although there are some designs without symmetry of revolution on the market, e.g. LENTIS MPlus (Oculentis), continuous change of optical power exhibited by the LSL is a new concept which could be used for ophthalmic applications.

Recent simulations and experimental results, performed with the help of the optical system of the artificial eye imitating the presbyopic human eye, show that the LSL light focusing properties make possible formation of images for objects located at a wide range of distances from 25–33 cm to infinity. These images exhibit consistently good quality with an acceptable contrast. [10–15] The main goal of the LSL design is then to provide homogeneous and fine vision for presbyopes through continuous extension of depth-of-field. Recently we presented the first psychophysical experiments that evaluated the ability of the LSL to compensate a lack of accommodation. Visual acuity of 11 subjects with drug-induced cycloplegia was measured using a simulator of monocular vision. [16]

This paper presents the results of the first clinical study, in which the LSL was used to correct vision of patients with natural presbyopia. The aim of this study is to characterize visual performance with LSL correction in comparison to uncorrected presbyopic eyes and stenopeic vision.

## Material and methods

The S1 Protocol was approved by the Bioethics Committee of the Military Institute of Medicine in Warsaw in April 2015 and adhered to the tenets of the Declaration of Helsinki. The only modification of the study was the resignation of the examination of younger patients (Groups I and II in the S1 Protocol), therefore the study included more patients from Group III. Written consent was obtained from all participants after they had been informed about the investigative character of the study and declared their willingness to participate.

**Fig 1:**
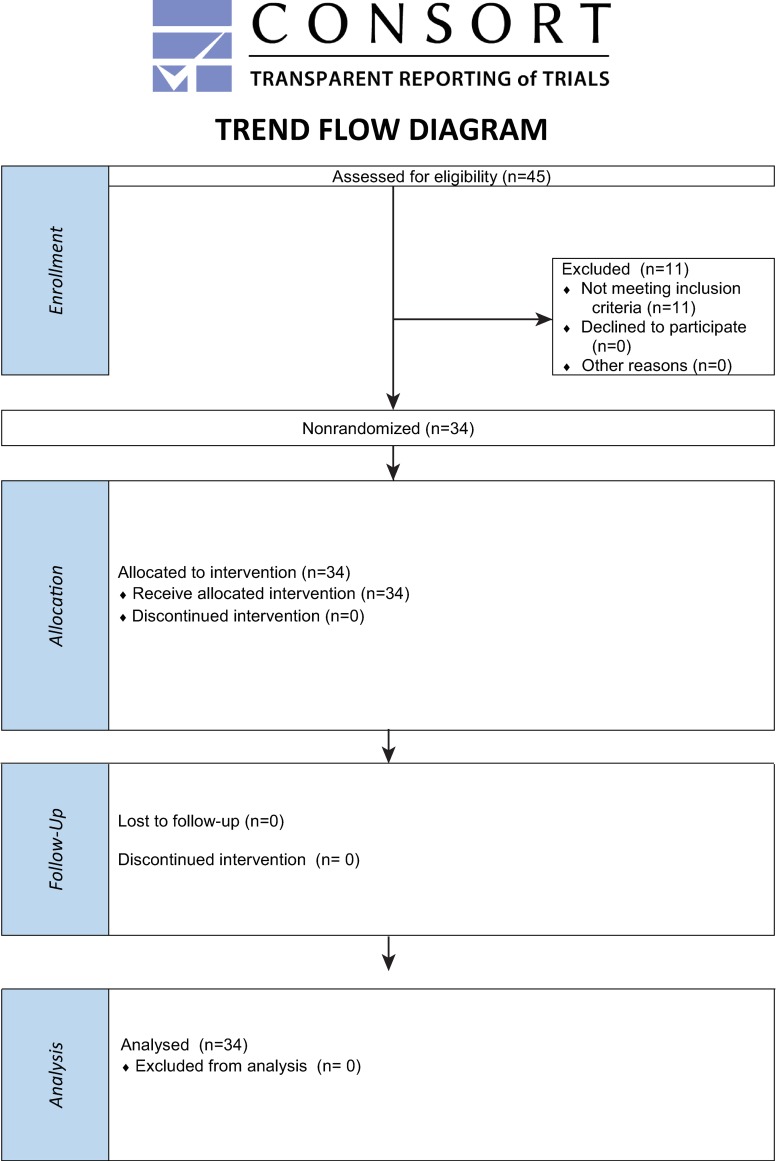
The flow chart of the trial according to the Transparent Reporting of Evaluations with Nonrandomized Designs (TREND).

The non-randomized recruitment process of participants among technical and administrational staff of Military Institute of Medicine in Warsaw, Poland started on April 2016 and lasted until April 2017. The whole trial for each patient covered single two hour session including recruiting, examination and data collection performed by ophthalmologists not involved in study design. There was no follow-up of examined patients. The study was registered at clinicaltrials.gov (NCT03716271) after the data was collected and total number of examined patients was achieved. Although the Department of Ophthalmology of the Military Institute of Medicine in Warsaw (Poland) has vast experience in the registration of prospective clinical trials, it was not registered prior to recruitment of patients, because it included a single examination of patients without further follow-up, and the authors did not realize that such registration is also required, which is the reason for the delay in registration of the study. The authors confirm that all ongoing and related trials for this intervention are registered. Details of each trial is described below and flow chart of the Transparent Reporting of Evaluations with Nonrandomized Designs (TREND) is shown in Fig 1.

The study included emmetropic or hyperopic eyes with a spherical error of maximum +1.75D and astigmatism of ≤ 0.5D. Corrected distance visual acuity (CDVA) had to be better than 0.1 logMAR (20/25) and at the same time contrast sensitivity (CS) could not be less than 1.9 logCS. Uncorrected near visual acuity was required to be worse than 0.4 logMAR (20/50), as a proof of presbyopia. Exclusion criteria contained: any history of ophthalmic surgeries, evidence of serious ocular or brain pathologies affecting visual acuity (VA) and clinically active inflammation.

Only dominant eyes, verified with the “hole-in-the-hand” test, [17] were tested in the study. Group of 45 potential subjects was recruited while visual outcomes of 34 presbyopic patients meeting inclusion criteria were taken for analyses. After recruitment and examination of the first five subjects, the standard deviation of differences in results for each planned paired comparison was calculated. The maximal value was 0.167 logMAR/logCS. The minimal clinically significant difference is equal to 0.1 logMAR/logCS. From Altman Nomogram it can be estimated that for such values it is necessary to recruit sample of at least 32 subjects to obtain power at level 0.8 for significance α = 0.01. We used the common Shapiro-Wilk test to check the normality of the data distribution. We received a negative result, therefore we used non-parametric tests. For limitation of order bias each subject performed trials in a random order without being informed about target of the study.

Patients were examined for VA and CS in three refractive conditions: reference trial (REF) which was carried out using circular aperture with diameter of 8 mm, STENO trial was performed through a pinhole limiting iris aperture diameter to 1.25 mm and LSL trial was made using the LSL of diameter of 8 mm set in a trial frame. Examinations were performed in natural light conditions (i.e. the natural pupil formed the additional aperture stop when using the REF condition).

Each visual performance trial consisted of nine tasks–seven VA examinations for defocus values (0.2 D, 0.5 D, 1.0 D, 1.5 D, 2.0 D, 2.5 D, 3.0 D) and two CS assessments with tasks for defocus 0.3 D and 2.5 D.

**Fig 2:**
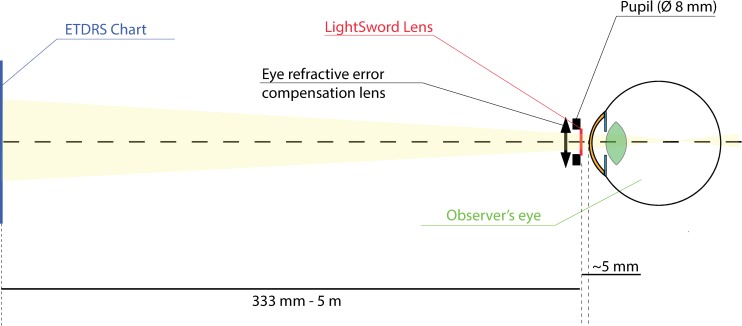
The optical setup of the subjective visual acuity test in Light Sword Lens (LSL) trial.

The CDVA was adjusted using Early Treatment Diabetic Retinopathy Study (ETDRS) [18] chart read from a distance of 5.0 m. Then different ETDRS charts were shown for assumed distances in consecutive tasks. All ETDRS charts were printed on high quality printers, properly scaled for appropriate distance (Fig 2). [18]

Contrast sensitivity tasks were performed with commercially available charts (Gima s.p. A., Gessate (Milano), Italy, Prod. Code 313317 and 31318) basing on Pelli-Robson method. The charts were designed for near and distant vision corresponding to defocus 2.5 D (0.4 m) and 0.3 D (3.0 m) and composed of 21 sets of triplets related to values from 2.0 to 0.0 logCS with a step of 0.1 logCS. [19] In order to compare CS at both distances +2.5 D lens in the 0.4 m task in REF trial was added.

**Fig 3:**
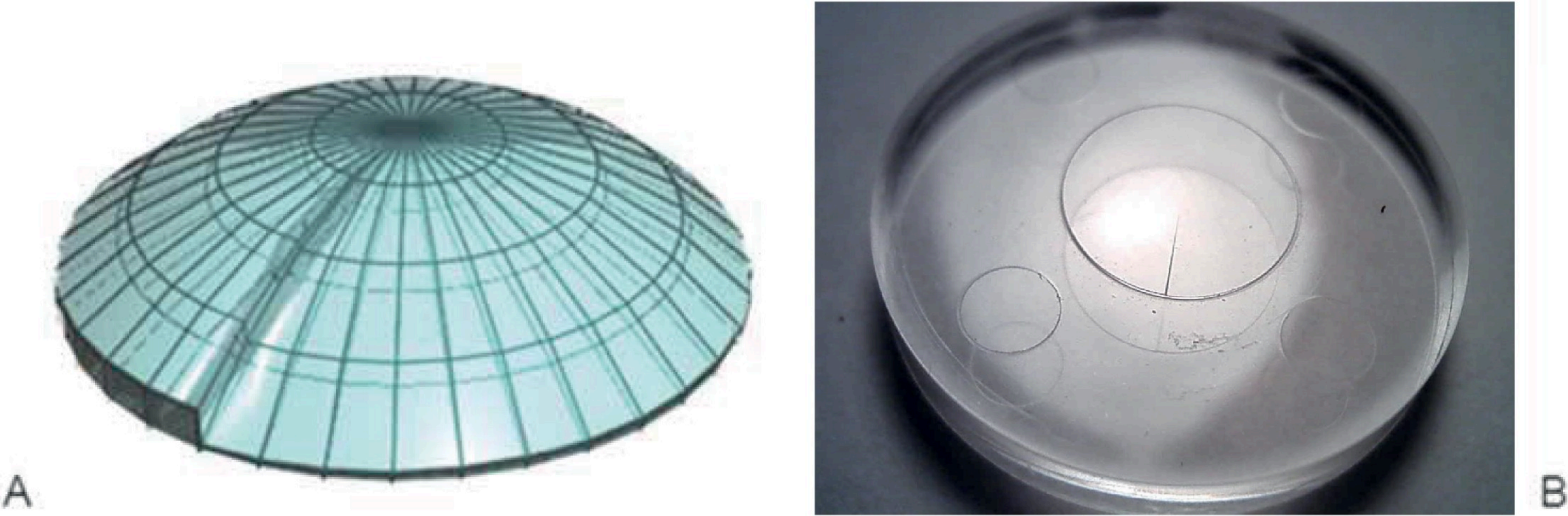
The Light Sword Lens (LSL). A–design visualization, B–fabricated lens.

Light Sword Lens has some range of optical power (in case of this experiment it was 0–3 D) distributed continuously along angular position of its own semi-diameter (Fig 3A). [15, 16] None of its cross-sections through the center is the same–each of them is associated with different optical power and therefore also with different shape. At 0°/360° angular position, maximal and minimal power meet each other causing characteristic sharp edge along one of semidiameters. In LSL trials optical element was placed into a trial frame at the vertex distance of 5 mm and this edge was oriented at 0° axis (Fig 2). For purpose of this study LSL was manufactured in a plano-convex form (Fig 3B).

Visual acuity in logMAR scale or contrast sensitivity in logCS scores were assigned based on the last line or last set with more than half correctly recognized optotypes, respectively. Luminance of ETDRS and CS charts surface was 200 (±20) cd/m^2^, while illuminance of the room was about 300 lx, which was measured with photometer (Seconic Digital Master.L-758DR). The ceiling effect for CS is acknowledged in the section “Discussion”.

### Data analysis methods

Friedman ANOVA and Nemenyi post-hoc tests were used for multiple comparisons. Verification of differences of two groups was conducted with Wilcoxon signed-rank test. Additionally as a measure of similarity of outcomes, particular effect sizes Z and their threshold Z_crit_ were presented. Test statistics Z can be treated as effect sizes of defocus in one trial as well as it can be interpreted as the impact of specific correction on visual performance for the specific defocus. [20]

*P*-value of α ≤ 0.05 was considered to be significant in Friedman ANOVA and Wilcoxon signed-rank test. In case of Nemenyi post-hoc tests corrections for number of cases were calculated, due to which the significance level was changed. For statistics comparing corresponding tasks outcomes in REF, STENO and LSL trials, P-value of α’ ≤ 0.0083 was considered to be significant. Whereas for statistics comparing VA results of different defocus within same trial, significance was reduced to α’ ≤ 0.0012. Calculations were performed with Statistica 10.0 Software.

## Results

There were 34 eyes of 34 subjects of mean age 54.6 years (min: 47 y.; max: 62 y.; SD: 4.5 y.) examined in this study. Fourteen participants were female and 20 right eyes were included. The median spherical correction was (+) 0.64 D (min: 0.00 D; max: 1.75 D; IQR: 1.00 D), whereas cylindrical (-) 0.06 D (min: 0.00 D; max: -0.50 D).

### Visual performance degradation with defocus–visual acuity

**Fig 4:**
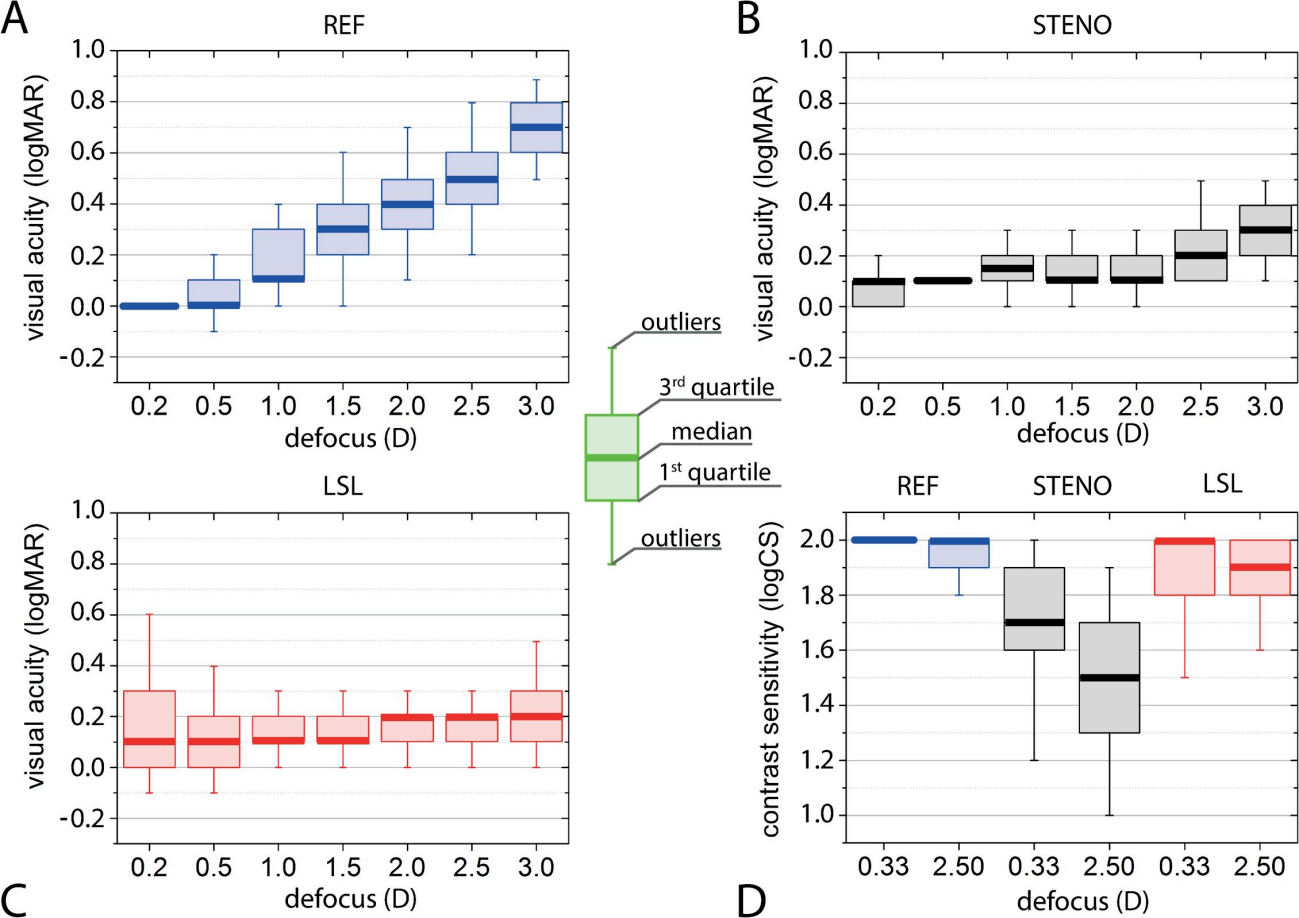
**Results of visual acuity** (charts A–C) **and contrast sensitivity** (chart D) **for different defocus values.** Blue marks are for the REF trial, black–for the STENO and red–for the LSL trial.

The REF trial, showed results consistent with the assumed characteristics for distant vision (VA: 0.2 D– 0.0 logMAR (20/20); CS: 0.3 D– 2.0 logCS). During defocusing, VA was constantly degraded with almost linear growth (χ^2^ = 197 *P* <0.001) (Table 1 and Fig 4A and S1 Dataset). Pairwise comparison of VA showed that REF trial was characterized by significant degradation starting from a defocus of 1.0 D (Z_1,34_ ≥ 3.48, *P*<0.001) (Table 1).

**Table 1:** Comparison between distant vision and each defocus in VA and CS tasks.

	**VA tasks**							**CS tasks**	
**Defocus:**	**0.2 D**	**0.5 D**	**1.0 D**	**1.5 D**	**2.0 D**	**2.5 D**	**3.0 D**	**0.3 D**	**2.5 D**
**Chart distance:**	**5.00 m**	**2.00 m**	**1.00 m**	**0.67 m**	**0.50 m**	**0.40 m**	**0.33 m**	**3.00 m**	**0.40 m**
**FRIEDMAN TESTS**	**PAIRWISE NEMENYI POST-HOC TESTS**							**WILCOXON SIGNED-RANK TESTS**	
**(all defocus values)**	**(for 0.2 D vs. following defocus values)**							**(for 0.3 D vs. 2.5 D)**	
N = 34, df = 6	N = 34, df = 1,							N = 34, df = 1	
α = 0.050	α = 0.001, Z_crit_ = 3.24							α = 0.050, Z_crit_ = 1.65	
**REF trial**									
	[logMAR]							[logCS]	
Q_1_	0.00	0.00	0.10	0.20	0.30	0.43	0.60	2.00	1.90
**Q**_**2**_	**0.00**	**0.00**	**0.10**	**0.30**	**0.40**	**0.50**	**0.70**	**2.00**	**2.00**
Q_3_	0.00	0.10	0.28	0.40	0.50	0.60	0.78	2.00	2.00
χ2 = 197	Z = 0.00	Z = 1.04	Z = 3.48	Z = 5.02	Z = 7.16	Z = 8.84	Z = 10.8	Z = 2.90	
P < 0.001	P = 1.000	P = 0.299	P < 0.001	P < 0.001	P < 0.001	P < 0.001	P < 0.001	P = 0.004	
**STENO trial**									
	[logMAR]							[logCS]	
Q_1_	0.00	0.10	0.10	0.10	0.10	0.13	0.20	1.60	1.30
**Q**_**2**_	**0.10**	**0.10**	**0.15**	**0.10**	**0.20**	**0.20**	**0.30**	**1.70**	**1.50**
Q_3_	0.10	0.10	0.20	0.20	0.20	0.30	0.38	1.90	1.68
χ2 = 129	Z = 0.00	Z = 1.18	Z = 3.87	Z = 3.34	Z = 5.05	Z = 6.74	Z = 8.50	Z = 4.48	
P < 0.001	P = 1.000	P = 0.238	P < 0.001	P < 0.001	P < 0.001	P < 0.001	P < 0.001	P< 0.001	
**LSL trial**									
	[logMAR]							[logCS]	
Q_1_	0.00	0.00	0.10	0.10	0.10	0.10	0.10	1.80	1.80
**Q**_**2**_	**0.10**	**0.10**	**0.10**	**0.10**	**0.10**	**0.20**	**0.20**	**2.00**	**1.90**
Q_3_	0.28	0.18	0.20	0.20	0.20	0.20	0.30	2.00	2.00
χ2 = 35.9	Z = 0.00	Z = 1.52	Z = 0.06	Z = 0.36	Z = 0.28	Z = 1.49	Z = 3.40	Z = 0.68	
P < 0.001	P = 1.000	P = 0.130	P = 0.955	P = 0.715	P = 0.779	P = 0.137	P < 0.001	P = 0.496	

Q1 / Q2 / Q3 –first /second (median) / third quartile, χ2 –Friedman test statistic (cumulative measure of differences), Z–Nemenyi or Wilcoxon test statistic (effect size), Zcrit: critical effect size. Significant differences are underlined.

The STENO trial exhibited a limited range of VA degradation (VA: 0.2 D– 0.1 logMAR (20/25); 3 D– 0.3 logMAR (20/40)), however similarly to REF trial, significant differences were observed for all near and intermediate distances till 1.0 m (χ^2^ = 129 *P* <0.001). This situation was associated with low deviation of results within the STENO group resulting from high repeatability of measurements. (Table 1 and Fig 4B and S1 Dataset)

In the case of LSL trial, VA varied only within a small range from 0.1 to 0.2 logMAR (20/25 to 20/32) in the full defocus range (Fig 4C and S1 Dataset). Although the differences within trial were significant, the test statistic χ^2^ was much lower than in the REF and STENO cases (χ^2^ = 35.9, *P* < 0.001) and significant degradation of performance was observed only at the nearest distance (Z = 3.40, *P* <0.001) (Table 1).

**Fig 5:**
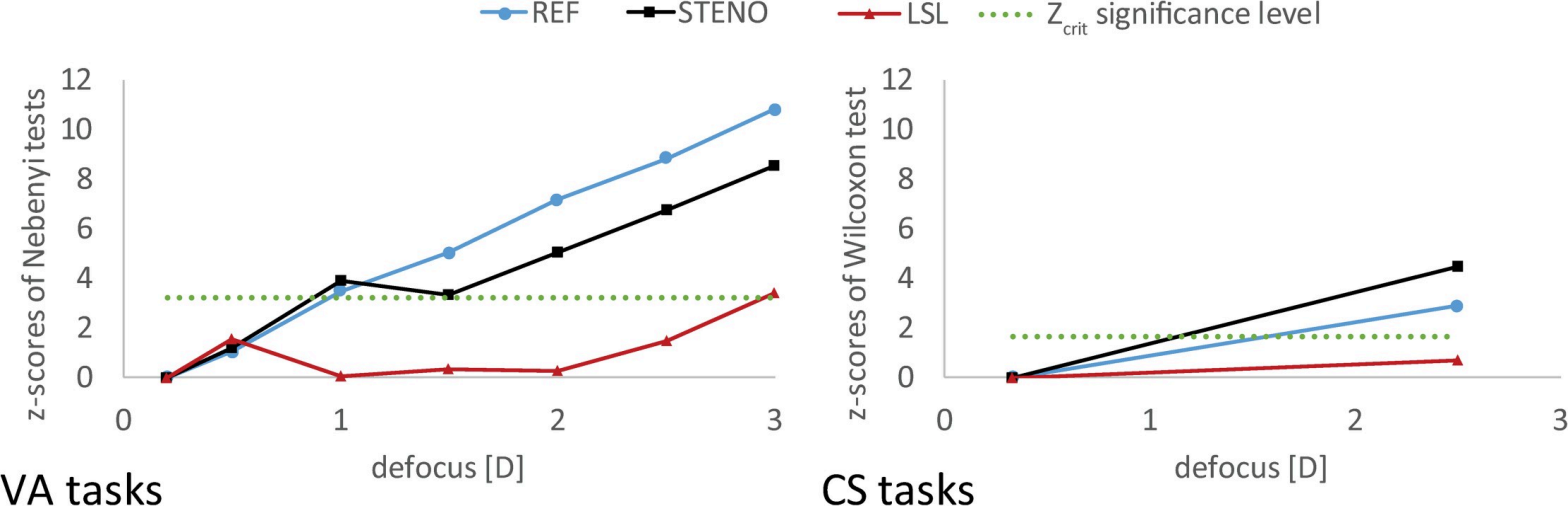
Effect sizes of defocus for each correction method (according to pairwise Nemenyi post-hoc tests and Wilcoxon signed ranks test presented in Table 1). Significant differences are located above Z_crit_ level.

The effect size for REF and STENO trials crossed threshold Z_crit_ = 3.24 (defined by the significance level α’ = 0.0012) marked in dotted line at a defocus of 1.0 D (Fig 5). The Z-score describing REF VA degradation grew almost linearly, exceeding 10 for near vision (defocus: 3.0 D; Z_1,34_ = 10.8). Z-scores corresponding to the STENO case were below 4 until the intermediate vision distance (defocus 1.5 D; Z_1,34_ = 3.3), and they grew in near vision (defocus 3.0 D; Z_1,34_ = 8.5). Z-scores obtained in the LSL task were smaller than 1.5, except the maximum value equal to 3.4, obtained for the near distance of 33 cm (3.0 D).

### Visual performance degradation with defocus–contrast sensitivity

In REF trial CS remained at a high level for both: distant (CS 0.3D – 2.0 logCS) and near vision (CS: 2.5 D– 2.0 logCS), though fine differences were indicated to be significant (Z = 2.90, P = 0.004) (Table 1 and Fig 4D and S1 Dataset).

Stable VA results in STENO trial were accompanied by a low CS performance for near (2.5 D– 1.5 logCS) as well as for distance (CS: 0.3 D– 1.7 logCS) vision (Table 1 and Fig 4D), S1 Dataset. CS outcomes showed significant variability (Z = 4.48, P < 0.001). The median of CS results for LSL trial decreases from far vision (2.0) to near vision (1.9) (Z = 0.68 _,_ *P* = 0.496) (Table 1 and Fig 4D and S1 Dataset). In the case of CS tasks, the assumed parameter α = 0.05 determined the threshold Z_crit_ = 1.65. Z -scores describing the difference between distant and near CS shown graphically in Fig 5, were equal to 4.5, 2.9 and 0.68 for the STENO, REF and LSL task cases, respectively.

### Comparison of defocused vision in different trials

**Fig 6:**
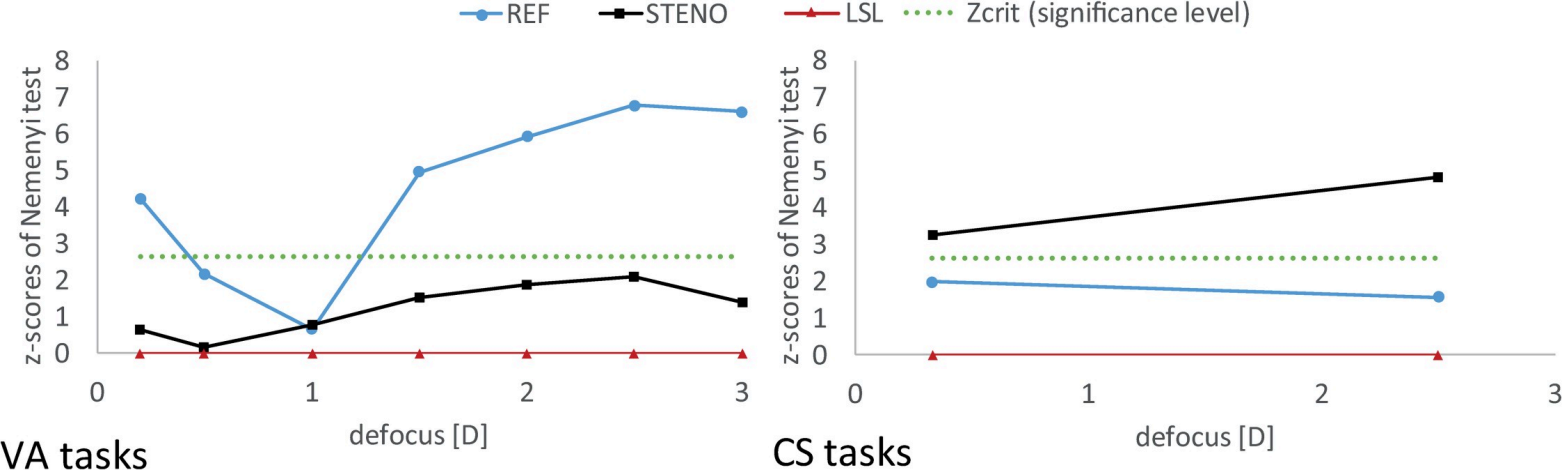
Effect sizes of correction methods for each defocus (according to pairwise Nemenyi post-hoc tests presented in Table 2). Significant differences are located above Z_crit_ level.

Visual acuity and CS examinations performed in REF, STENO and LSL trials with the same defocus were significantly different (χ^2^ ≥ 9.61, *P* ≤ 0.008). (Table 2), besides VA tasks corresponding to the distance of 1.0 m (defocus 1.0 D) (χ^2^ = 1.02, *P* = 0.600), LSL outcomes for each defocus were compared with other trials for the same distances with Nemenyi post-hoc tests. The significance level was assumed to be α = 0.0083 associated with Z_crit_ = 2.64. Visual acuity measured in the REF and LSL trials differed significantly for all defocuses (Z_1,34_ > 2.64_,_ *P* < 0.001), except 0.5 D and 1.0 D (Z_1,34_ ≤ 2.18, *P* ≥ 0.029). Outcomes of VA in case of LSL and STENO on the other hand, were comparable for all defocuses (Z_1,34_ ≤ 2.12, *P* ≥ 0.034). While CS results were similar for LSL and REF (Z_1,34_ ≤ 2.00, *P* ≥ 0.045), significant differences for CS were found between the LSL and the STENO (Z_1,34_ ≥ 3.27, *P* < 0.001). The comparisons LSL vs. REF and LSL vs. STENO were illustrated by plots given in Fig 6, where Z_crit_ = 2.64 was marked by the dotted line.

**Table 2:** Results of comparison between LSL vs. REF and LSL vs. STENO, VA and CS examination for each defocus.

		Friedman tests	Nemenyi post-hoc tests	
		N = 34, df = 2, α = 0.050	N = 34, df = 1, α = 0.0083, Z_crit_ = 2.64	
Defocus (chart distance)		REF vs. STENO vs. LSL	LSL vs	
			REF	STENO
**VA tasks**	0.2 D (5.00 m)	χ^2^ = 27.3, *p* < 0.001	Z = 4.24, *p* < 0.001	Z = 0.67, *p* = 0.505
	0.5 D (2.00 m)	χ^2^ = 9.61, *p* = 0.008	Z = 2.18, *p* = 0.029	Z = 0.18, *p* = 0.856
	1.0 D (1.00 m)	χ^2^ = 1.02, *p* = 0.600	Z = 0.67, *p* = 0.505	Z = 0.79, *p* = 0.431
	1.5 D (0.67 m)	χ^2^ = 33.0, *p* < 0.001	Z = 4.97, *p* < 0.001	Z = 1.52, *p* = 0.130
	2.0 D (0.50 m)	χ^2^ = 45.6, *p* < 0.001	Z = 5.94, *p* < 0.001	Z = 1.88, *p* = 0.060
	2.5 D (0.40 m)	χ^2^ = 52.1, *p* < 0.001	Z = 6.79, p < 0.001	Z = 2.12, *p* = 0.034
	3.0 D (0.33 m)	χ^2^ = 53.7, *p* < 0.001	Z = 6.61, *p* < 0.001	Z = 1.39, *p* = 0.163
**CS tasks**	0.3 D (3.00 m)	χ^2^ = 41.5, *p* < 0.001	Z = 2.00, *p* = 0.045	Z = 3.27, *p* < 0.001
	2.5 D (0.40 m)	χ^2^ = 52.6, *p* < 0.001	Z = 1.58, p = 0.115	Z = 4.85, p < 0.001

SD–standard deviation, χ2 –Friedman test statistic (cumulative measure of differences), Z–Nemenyi test statistic (effect size), Z_crit_: critical effect size. Significant differences are underlined.

## Discussion

In this paper visual outcomes of the LSL as a new method for presbyopia correction of 34 recruited subjects were presented. Their uncorrected presbyopic vision (REF) exhibited substantial degradation of VA with defocus (Figs 4A and 5 and Table 1: VA tasks) while corrected with LSL near and distant vision were characterized by acceptable VA and full CS (Fig 4D and Table 1: CS tasks). In order to better illustrate properties of the LSL, LSL results were confronted to STENO task outcomes, in which pupil is artificially limited by a small aperture. Such pinhole similarly to the camera obscura, realizes imaging with the extended depth of field (EDOF) with substantially suppressed contrast. These well-known features of pinhole imaging were well illustrated in (Fig 4B and 4D and Table 1). According to obtained results, the LSL provided uniform VA in whole range of investigated defocus with medians not higher than 0.2 logMAR (20/32) (Fig 4C and 5 and Table 1: VA tasks). Moreover correction by the LSL was accompanied by high quality CS in distant and near vision (Fig 4D and Table 1: CS tasks). Quality of VA was similar to that obtained in STENO (Table 2: VA tasks, Fig 6) and contrast vision exhibited coincidence with CS obtained in REF (Table 2: CS tasks, Fig 6).

A comparison of the studied tasks permits the interpretation that LSL provided significantly better VA for defocus values greater than 1 D compared to uncorrected presbyopic vision (REF). Although any differences within the 0.5–1.0 D range could have arisen from sampling error (Table 2: LS vs. REF), the LSL performed worse in distant vision (0.2 D). This results coincide with former subjective experiments in a visual simulator, where the LSL was used for compensation of artificially paralyzed accommodation [16] in a group of young subjects. The problem with worse distant vision performance demands partial redesign of the element. The profile of the LSL is defined by the linear angular function, [14, 15] which can be modified in order to improve distant vision results, preserving angular modulation of the optical power. Moreover, due to technological reasons, a redesign should lead to smoothing the sharp edge between the minimum and maximum optical power. Some preliminary attempts in this direction seem promising. [21] While commonly used multifocal lenses have two or more annular areas of disparate power, its functionality can be limited through aperture diameter changes cutting off some parts of active surface. Contrary to that, the LSL geometry of light focusing is not limited by any circular aperture and characterized by whole range of optical power regardless of iris diameter. The LSL shoud be placed ultimately in an entrance pupil of the eye in a form of contact lens (CL) or IOL not available yet due to technological difficulties. The used prototype of the LSL introduces a residual dependence on correction power affecting different visual field parts in peripheral vision. Such effect would not exist in the cases of CL or IOL. In our study this problem was minimized by careful alignment of the lens and placing charts in the central part of visual field.

It should be mentioned that LSL retinal point spread function has no rotational symmetry with the shape and position depending on focusing distance. However this effect doesn’t seem to have affected the results much in the present case but could have significance in some visual tasks, what should be checked after manufacturing the LSL in a form of CL or IOL.

In CS assessment Pelli-Robson method was chosen because of their recognition-based character, which is more important for near vision and more comparable to visual acuity tests. Used commercial charts had maximal score 2.0 logCS what was below contrast sensitivity threshold of all subjects. This value is fully sufficient for high quality vision therefore higher values have low clinical implications. Such limitation created however “ceiling effect” perturbing some statistical analysis. For example, due to very low deviation of results in CS distant tasks of the REF trial, differences between distance and near contrast vision was indicated as significant, despite the same median. According to obtained results, the LSL contrast vision was better than in the case of stenopaeia and insignificantly worse than CS corresponding to the REF corrected presbyopic vision. Such insignificance could also be associated with this “ceiling effect” but even then LSL contrast vision loss related to functional level was relatively low.

Among the presented results P-values as well as effect sizes (Z) were shown. P-value can be interpreted as qualitative factor determining significance of measured variances, while the effect sizes are quantitative parameters, expressing magnitude of the obtained differences. In the interpretation of homogeneity degree of the whole trial results in turn, the Friedman test statistic χ2 was a substantial parameter, corresponding to a cumulative measure of variation between cases. All these parameters were particularly useful, because they allowed to quantify obtained differences and to define the significance levels using proper probability distributions. Therefore, Zcrit corresponding to α or α’ were calculated and for better visualization of obtained differences Z-scores were plotted in charts comparing distant and defocused vision in order to clearly show differences between various refractive variants.

The obtained results showed that the LSL effectively enables EDOF vision well beyond the natural depth of field of the human eye [22–25] and thereby provides an efficient remedy for limitations of visual acuity caused by presbyopia. The main goal of this paper was to prove feasibility of LSL correction. The presented pilot clinical study encourages follow up research analyzing applicability of the LSL as the method of presbyopia correction. Further investigations will require additional measurements analyzing such problems as decentration, tilt, rotation, imperfections of shape and many other factors that should be checked-out before the full clinical study. However reliable investigation can be performed with the LSL in a form of CLs or IOLs, which are unavailable yet and are being at the stage of preliminary elaboration.

## Supporting information

S1 Protocol Study protocol approved by bioethical commission in Polish and its translation to English. (PDF)

S1 Dataset Source data obtained in the study. (XLSX)

S1 Checklist TREND statement checklist. (PDF)

## References

[1] United Nations. Dept of Economic and Social Affairs. Population Division. World Population Ageing 2015. New York; UN; 2015 Available from: http://www.un.org/en/development/desa/population/publications/pdf/ageing/WPA2015_Report.pdf [Accessed: 14 Jan 2019].

[2] Holden BA, Fricke TR, Ho SM, Wong R, Schlenther G, Cronjé S et al Global vision impairment due to uncorrected presbyopia. Arch Ophthalmol. 2008;126:1731–1739. 10.1001/archopht.126.12.1731 19064856

[3] Charman WN. Developments in the correction of presbyopia I: spectacle and contact lenses. Ophthalmic Physiol Opt. 2014;34:8–29. 10.1111/opo.12091 24205890

[4] Rocha K. Extended Depth of Focus IOLs: The Next Chapter in Refractive Technology? J Refract Surg. 2017;33:146–149. 10.3928/1081597X-20170217-01 28264127

[5] Davison JA, Simpson MJ. History and development of the apodized diffractive intraocular lens. J Cataract Refract Surg. 2006;32:849–858. 10.1016/j.jcrs.2006.02.006 16765805

[6] Żelichowska B, Rękas M, Stankiewicz A, Cerviño A, Montés-Micó R. Apodized diffractive versus refractive multifocal intraocular lenses: Optical and visual evaluation. J Cataract Refract Surg. 2008;34:2036–2042. 10.1016/j.jcrs.2008.06.045 19027556

[7] Kołodziejczyk A, Bará S, Jaroszewicz Z, Sypek M. The light sword optical element—a new diffraction structure with extended depth of focus. J Mod Opt. 1990;37:1283–1286. 10.1080/09500349014551431

[8] Mikuła G, Jaroszewicz Z, Kołodziejczyk A, Petelczyc K, Sypek M. Imaging with extended focal depth by means of lenses with radial and angular modulation. Opt Express. 2007;15:9184–9193. 10.1364/OE.15.009184 19547260

[9] Ares García J, Bará S, Gomez García M, Jaroszewicz Z, Kolodziejczyk A, Petelczyc K. Imaging with extended focal depth by means of the refractive light sword optical element. Opt Express. 2008;16:18371–18378. 10.1364/OE.16.018371 18958115

[10] Petelczyc K, Ares Garcia J, Bará S, Gomez Garcia M, Jaroszewicz Z, Kołodziejczyk A. et al Presbyopia compensation with a light sword optical element of a variable diameter. Photonics Lett Pol. 2009;1:55–57. Available from: http://photonics.pl/PLP/index.php/letters/article/view/1-19/21 [Accessed: 14 Jan 2019].

[11] Galego AA, Bara S, Jaroszewicz Z, Kołodziejczyk A. Visual Strehl performance of intraocular lens designs with extended depth of focus. Optom Vis Sci. 2012;89:1702–1707. 10.1097/OPX.0b013e3182775e1a 23190714

[12] Petelczyc K, García JA, Bará S, Jaroszewicz Z, Kakarenko K, Kolodziejczyk A et al Strehl ratios characterizing optical elements designed for presbyopia compensation. Opt Express. 2011;19:8693–8699. 10.1364/OE.19.008693 21643121

[13] Petelczyc K, Bará S, Ciro López A, Jaroszewicz Z, Kakarenko K, Kołodziejczyk A et al Contrast transfer properties of the light sword optical element designed for presbyopia compensation. J Eur Opt Soc Rapid Publ. 2011;6:11053, 10.2971/JEOS.2011.11053

[14] Petelczyc K, Bará S, Lopez AC, Jaroszewicz Z, Kakarenko K, Kolodziejczyk A et al Imaging properties of the light sword optical element used as a contact lens in a presbyopic eye model. Opt Express. 2011;19:25602–25616. 10.1364/OE.19.025602 22273953

[15] Kakarenko K, Ducin I, Grabowiecki K, Jaroszewicz Z, Kolodziejczyk A, Mira-Agudelo A. Assessment of imaging with extended depth-of-field by means of the light sword lens in terms of visual acuity scale. Biomed Opt Express. 2015;6:1738–1748. 10.1364/BOE.6.001738 PMCID: PMC4467699 26137376 PMC4467699

[16] Mira-Agudelo A, Torres-Sepúlveda W, Barrera JF, Henao R, Blocki N, Petelczyc K et al Compensation of presbyopia with the Light Sword Lens. Invest Ophthalmol Vis Sci. 2016;57:6870–6877. 10.1167/iovs.16-19409 28002561

[17] Steinman S, Steinman BA, Garzia RP. Foundations of Binocular Vision: A Clinical Perspective. New York, NY US: McGraw-Hill, 2000.

[18] Colenbrander, A. Visual standards: aspects and ranges of vision loss with emphasis on population surveys. Report for the International Council of Ophthalmology. 2002;1–33. Available from: http://www.icoph.org/downloads/visualstandardsreport.pdf [Accessed: 14 Jan 2019].

[19] Pelli DG, Robson JG. The design of a new letter chart for measuring contrast sensitivity. Clinical Vision Sciences. 1988;2(3):187–199. Available from: https://psych.nyu.edu/pelli/pubs/pelli1988chart.pdf [Accessed: 14 Jan 2019].

[20] Rosenthal R. Parametric measures of effect size In: Cooper H, Hedges LV, editors. The handbook of research synthesis. New York, NY US: Russell Sage Foundation 1994 pp. 231–244.

[21] Kakarenko K, Ducin I, Jaroszewicz Z, Kołodziejczyk A, Petelczyc K, Stompor A, et al: "Optical elements with extended depth of focus and arbitrary distribution of intensity along the focal segment obtained by angular modulation of the optical power" J Phys Conf Ser 2015;605:012012 10.1088/1742-6596/605/1/012012

[22] Green DG, Powers MK, Banks MS. Depth of focus, eye size and visual acuity. Vision. 1980;20:827–835. 10.1016/0042-6989(80)90063-2 7467137

[23] Wang B, Ciuffreda KJ. Depth-of-Focus of the Human Eye: Theory and Clinical Implications. Surv Ophthalmol. 2006;51:75–85. 10.1016/j.survophthal.2005.11.003 16414364

[24] Atchison DA, Charman WN, Woods RL. Subjective depth-of-focus of the eye. Optom Vis Sci. 1997;74:511–520. 10.1097/00006324-199707000-00019 9293519

[25] Legge GE, Mullen KT, Woo GC, Campbell FW. Tolerance to visual defocus. J. Opt Soc Am A. 1987;4:851–863. 10.1364/JOSAA.4.000851 3598739

