# Supplementary material for: The Light Sword Lens - A novel method of presbyopia compensation: Pilot clinical study
Source: PLoS One. 2019 Feb 4;14(2):e0211823. doi: 10.1371/journal.pone.0211823 (PMC6361462; doi:10.1371/journal.pone.0211823)
Supplement: S1 Protocol — (PDF) [file pone.0211823.s001.pdf]

Wypełnia Komisja: Data zgłoszenia projektu: 10.04.2015 Numer projektu: 610

SEKRETARZ KOMISJI BIOETYCZNEJ WIM mgr Ewa JEDRZEJCZAK

PROJEKT BADAŃ
zgłoszony do Komisji Bioetycznej Wojskowego Instytutu Medycznego

1. Tytuł projektu:

Parametry jakościowe widzenia ze sztucznie zwiększoną głębią ostrości

2. **Autor/Kierownik projektu badawczego (imię i nazwisko, tytuł naukowy, specjalizacja)**:

płk prof. dr hab. n. med. Marek Rękas, specjalista chorób oczu

3. Członkowie zespołu badawczego (imię, nazwisko, tytuł naukowy, stanowisko służbowe, specjalizacja):

1. płk prof. dr hab. n. med. Marek Rękas, specjalista chorób oczu, kierownik Kliniki Okulistyki WIM
2. mjr lek. Rafał Pawlik, starszy asystent Kliniki Okulistyki WIM
3. dr inż. Krzysztof Petelczyc- Politechnika Warszawska - Wydział Fizyk
4. prof. dr hab. inż. Andrzej Kołodziejczyk - Politechnika Warszawska - Wydział Fizyk
5. mgr inż. Aleksandra Składowska - Politechnika Warszawska - Wydział Fizyk
6. mgr inż. Karol Kakarenko - Politechnika Warszawska - Wydział Fizyk
7. mgr inż. Izabela Ducin - Politechnika Warszawska - Wydział Fizyk

4. Kierownik jednostki organizacyjnej zgłaszającej projekt:

płk prof. dr hab. n. med. Marek Rękas, specjalista chorób oczu

5. Informacja o badaniach (w oddzielnym dokumencie wg poniższych punktów):

5.1 założenia i cel pracy (kluczowe pozycje piśmiennictwa)

Celem pracy jest sprawdzenie wpływu zastosowania soczewki sztucznie zwiększającej głębię ostrości widzenia na właściwości percepcyjne wzroku w warunkach rozogniskowania.

5.2 opis rodzaju badania (faza I, II, III itd.), grupa I-IV (np. studium pilotowe, studium z grupą kontrolną itp.)

Badanie pilotażowe typu seria przypadków

5.3 badani – wiek, płeć, stan zdrowia

Grupa I – osoby w wieku 20-30 lat
Grupa II – osoby w wieku 45-60 lat
Grupa III – osoby w wieku 56 – 70 lat
Osoby bez zaburzeń świadomości, dowolnej płci.

5.4 liczebność grupy badanej oraz kryteria włączenia do badań i wykluczenia z badań przed rozpoczęciem lub w trakcie

Grupa I: 3-5 osób.
Grupa II: 5-10 osób
Grupa III: 5-10 osób
Kryterium włączenia;

- wiek zgodny opisem grupy
- w przypadku grup II utrata akomodacji wymagająca korekcji powyżej 1D
- w przypadku grupy III utrata akomodacji wymagająca korekcji powyżej 2,5D

Kryterium wykluczenia:

- zaćma
- przebyta operacja zastąpienia naturalnych soczewek poprzez sztuczne soczewki wewnątrzgałkowe,
- widzenie dalekie po korekcji konwencjonalnej poniżej 1,0 w skali Snellena.
- zaburzenia psychiczne

5.5 przewidywany okres prowadzenia badań

12 miesięcy

5.6 opis produktów, które mają być zastosowane oraz sposób, dawka i schemat dawkowania badanego produktu kontrolnego

Wykorzystanie soczewek typu miecz świetlny, które sztucznie zwiększają głębię ostrości widzenia. Soczewki są objętościowymi refrakcyjnymi elementami optycznymi o krzywiźnie powierzchni zewnętrznej niesymetrycznej obrotowo. Zastosowane elementy wykonane z przezroczystego szkła akrylowego (PMMA) będą miały kształt płasko-wypukły. Ich idea działania polega na jednoczesnej korekcji oka mocą optyczną z całego zakresu zapewniającego widzenie funkcjonalne. W badaniach użyta zostanie soczewka

przeznaczona dla miarowego oka starczowzrocznego pozbawionego całkowicie akomodacji o zakresie mocy optycznych równy 0-3 dioptrie. Promień krzywizny elementu w sposób ciągły zmniejsza się wraz z obrotem przekroju od wartości odpowiadającej soczewce 0 dioptrii (płytce płasko-równoleglej) do profilu zgodnego z soczewką o maksymalnej mocy optycznej 3 dioptrie. (rys. 1). Taki kształt elementu skutkuje powstaniem ostrej krawędzi na styku minimalnej i maksymalnej mocy optycznej (rys. 1A - krawędź AB). Jej wysokość nie przekracza 50 μm. Średnica czynna stosowanej soczewki będzie wynosić 8 mm, zaś całego elementu 20 mm. Wraz z elementem zostanie zastosowana przesłona o średnicy 8 mm.

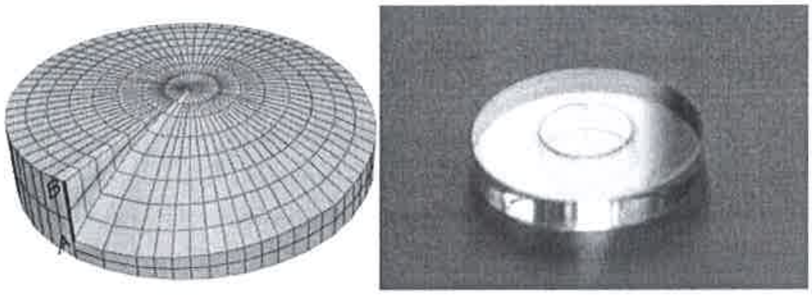

A B

Rys. 1.Kształt soczewki typu miecz świetlny (A - model, B - wykonana soczewka).

Idea soczewki wymaga jednoczesnego działania całej jej powierzchni. W praktyce oznacza to konieczność maksymalnego zbliżenia jej do źrenicy oka. Ponieważ badany model wykonany jest z materiału nie odpowiadającego wymogom soczewek kontaktowych w badaniu zostanie on umocowany w oprawkach okulistycznych w specjalnym mocowaniu zbliżającym soczewkę korekcyjną na odległość 5 mm od wierzchołka rogówki (rys. 2).

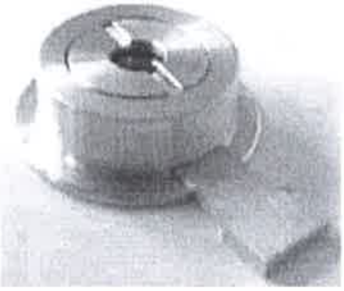

Rys. 2.Mocowanie soczewki do włożenia a obrawę okulistyczną.

Przez cały czas trwania badania wykorzystywane będą także konwencjonalne soczewki korekcyjne i pinhole okulistyczne. Przy próbach bez użycia soczewki "miecz świetlny" zastosowane zostanie to samo mocowanie, lecz puste.

5.7 metodyka badań (dokładnie)

Badani bo wywiadzie okulistycznym w zakresie spełnienia kryteriów włączenia i wykluczenia zostaną poddani badaniu przy pomocy autorefraktometru w celu orientacyjnego określenia wady refrakcyjnej oraz zakresu akomdacji. Następnie badanie zostanie powtórzone w oparciu o subiektywne odczucia badanego przy użyciu tablic okulistycznych do dali. Po dobraniu korekcji sferycznej i cylindrycznej pacjenci zostaną przebadani w celu określenia jakości widzenia za pomocą tablic okulistycznych zaprojektowanych dla odległości o rozogniskowaniu względem widzenia dalekiego w kierunku bliży w zakresie 0-3D. W następnej kolejności badanie zostanie powtórzone z wykorzystaniem soczewki miecz świetlny oraz pinholi okulistycznej. Ostatnią fazą

badania będzie wywiad dotyczący odczuć badanego, szczególnie w zakresie komfortu widzenia. Czas trwania sesji eksperymentalnej nie przekroczy 2 godzin.

5.8 charakter i stopień wszystkich znanych i przewidywanych zagrożeń (np. niepożądane efekty) **i korzyści dla zdrowia i życia badanych oraz wartości poznawczych i praktycznych związanych z prowadzeniem badań**

Nie przewiduje się zagrożeń związanych z prowadzeniem badań, przewiduje się nieznaczne przejściowe pogorszenie widzenia kontrastu w trakcie badania (podczas korekcji mieczem świetlnym) bez następstw po zakończeniu badania.
Stosowana soczewka typu miecz świetlny poprzez sztuczne zwiększenie głębi ostrości widzenia powinna zapewnić ostrość widzenia odpowiadającą widzeniu normalnemu na całym zakresie widzenia normalnego (0D-3D) co może stanowić skuteczną metodę kompensacji starczowzroczności.

5.9 proponowana ocena wyników badań i metod statystycznych, które mają być użyte do badań

Po wykonaniu badania dla każdej linii z tablic okulistycznych zostanie wyznaczony odsetek badanych osiągających dany rząd jakości widzenia (VA). Po wykreśleniu zależności tych wartości procentowych od kolejnych wartości VA dopasowana zostanie krzywa logistyczna i wyznaczony próg będący wartością VA linii, którą średnio poprawnie rozpoznało 50% badanych oraz niepewność związana z nachyleniem zależności w tym punkcie. Końcowym wynikiem badania będzie zależność progowych wartości VA od rozogniskowania wzroku przed i po korekcji soczewką typu miecz świetlny oraz dla widzenia przez pinholę okulistyczną.

5.10 w przypadku badania wieloośrodkowego informacje o całkowitym zasięgu badania

Badania prowadzone na terenie Wojskowego Instytutu Medycznego i Wydziału Fizyki Politechniki Warszawskiej.

5.11 wzór formularza „Świadomej zgody pacjenta", a w przypadku bardziej złożonych badań dodatkowo „Informacja dla pacjenta" zawierająca pełny i zrozumiały dla chorego opis badania i jego możliwych korzystnych i niekorzystnych skutków dla niego

Wyrażam zgodę na udział w eksperymencie dotyczącym innowacyjnej metody korekcji widzenia za pomocą elementów optycznych sztucznie zwiększających głębię ostrości widzenia. Jestem świadomy, że w trakcie badania moim zadaniem będzie rozpoznawanie optotypów na tablicach testowych. W trakcie wykonywania zadania będę patrzył przez różne elementy optyczne umieszczone w oprawkach okulistycznych.

5.1 **formułka:**

Oświadczamy, że jesteśmy świadomi, że wydanie zgody na badania przez Komisję Bioetyczną nie zwalnia nas od odpowiedzialności lekarskiej na zasadach ogólnych za zdrowie i życie powierzonych naszej opiece chorych.
Jednocześnie oświadczamy, że zapoznaliśmy się i zobowiązujemy się do przestrzegania „Zasad prawidłowego prowadzenia badań klinicznych produktów medycznych w krajach Wspólnoty Europejskiej".

KIEROWNIK KLINIKI [illegible]
[illegible]ntralnego Szpitala Klini[illegible]
Wojskowego Instytutu M[illegible]
prof. dr hab. n. med. [illegible]

Kierownik projektu badawczego: Marek [illegible] .... (Imię i nazwisko) .... (Podpis z pieczątką)

Członkowie zespołu badawczego:

Krzysztof Petelczyc .... (Imię i nazwisko) .... (Podpis)

Andrzej Kołodziejczyk .... (Imię i nazwisko) .... (Podpis)

Rafał Pawlik .... (Imię i nazwisko) .... (Podpis)

Izabela [illegible] .... (Imię i nazwisko) .... (Podpis)

Karol [illegible] .... (Imię i nazwisko) .... (Podpis)

Aleksandra [illegible] .... (Imię i nazwisko) .... (Podpis)

.... (Imię i nazwisko) .... (Podpis)

.... (Imię i nazwisko) .... (Podpis)

PROJECT OF THE TRIAL
notified to the Bioethical Commission
of the Military Institute of Medicine

1. **Title of the project:**

Quality parameters of vision with artificially increased depth of field

2. **Author / Head of the research team (name** and **surname, scientific title, specialization):**

Col. Prof. Marek Rękas PhD MD, specialist in Ophthalmology

3. **Members of the research team (name, surname, academic title, position, specialization):**
   1. Col. Prof. Marek Rękas PhD MD, specialist in Ophthalmology, head of the Department of Ophthalmology MIM
   2. Mjr Rafał Pawlik MD, senior assistant at the Ophthalmology Clinic WIM
   3. Krzysztof Petelczyc PhD Eng. - Warsaw University of Technology - Faculty of Physics
   4. Prof. Andrzej Kołodziejczyk PhD, Eng. - Warsaw University of Technology - Faculty of Physics
   5. Aleksandra Składowska MSc.- Warsaw University of Technology - Faculty of Physics
   6. Karol Kakarenko MSc.- Warsaw University of Technology - Faculty of Physics
   7. Izabela Ducin MSc.- Warsaw University of Technology - Faculty of Physicis

4. **Head of the reporting organizational unit:**

Col. Prof. Marek Rękas PhD MD, specialist in eye diseases

5. **Information about research (in a separate document according to the following points):**

5.1 Assumptions and purpose of the work (key positions of the literature)

The aim of the work is to check the effect of the use of a lens artificially increasing the depth of visual acuity on the perceptual properties of vision in defocus conditions.

5.2 Description of the type of study (phase I, II, III, etc.), group I-IV (e.g. pilot study, study with a control group etc.)

Pilot series case study

5.3 Subjects - age, gender, health condition

Group I - people aged 20-30.
Group II - people aged 45-60.
Group III - people aged 56-70
Persons without consciousness disorders, of any gender.

5.4 The size of the study group and the criteria for inclusion in the study and exclusion from the study from the start or during it

Group I: 3-5 people.

Group II: 5-10 people
Group III: 5-10 people

Inclusion criteria:

- age consistent description group
- in the case of groups II, loss of accommodation requiring correction above ID
- in case group III loss accommodation demanding correction above 2.5D

Criterion exclusion:

- cataract
- an operation of replacing natural lenses through artificial intraocular lenses,
- distant vision after conventional correction less than 1.0 on a scale Snellen.
- mental disorders

5.5 Expected research period

12 months

5.6 Description of the products to be used and the method, dose and dosing regimen of the product to be tested control

The use of Light Sword Lenses, which artificially increase the depth of field of vision. Lenses are volumetric refractive optical elements with a curvature of the outer surface rotationally asymmetric. The elements made of transparent acrylic glass (PMMA) will have a plano-convex shape. The idea of their action is based on the simultaneous correction of the eye with optical power from the entire range ensuring functional vision. The lens with an optical power range of 0-3 diopters, which will be used in the tests was designed for a presbyopic eye without any accommodation. The radius of curvature of the element is constantly decreasing with the rotation of the half-diameter cross-section from the shape corresponding to the 0 diopter (flat plate) to the corresponding lens profile with a maximum optical power of 3 diopters. (Fig. 1). This shape of the element results in a sharp edge at the minimum and maximum optical power (Fig. 1A - edge AB). Its height does not exceed 50 μm. The active diameter of the used lens will be 8 mm and the entire element diameter will be equal to 20 mm. A diaphragm with a diameter of 8 m will be used with the element =.

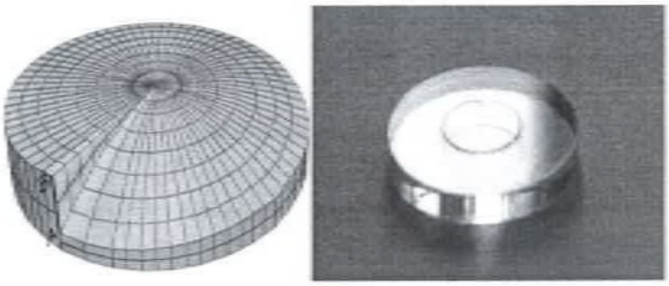

Fig. I. Light Sword Lens shape (A – model, B – manufactured lens)

The idea of a lens requires simultaneous action of its entire surface. In practice, this means the need to bring it close to the pupil of the eye. Because the tested model is made of material that does not meet the requirements of contact lenses, it will be fixed in

ophthalmic frames in a special fixture approaching the corrective lens at a distance of 5 mm from the top of the cornea (Fig. 2).

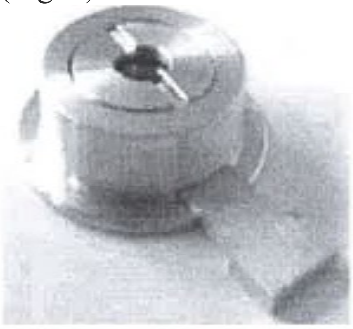

Fig . 2 . Fixing the lens for insertion into the ophthalmic frames .

Throughout the course of the study, conventional corrective lenses and the pinhole will also be used. For attempts without the use of a light sword lens, the same fixing will be used, but empty.

5.7 Research methodology (exactly)

The respondents, after the ophthalmic interview in the scope of meeting the inclusion and exclusion criteria, will be examined using the autorefractometer for the indicative determination of the refractive error and the range of accommodation. Then, the test will be repeated based on the subjective feelings of the subject using the distance charts. After selecting the spherical and cylindrical correction, the patients will be tested to determine the quality of vision using optometric charts designed for a distant vision with respect to near vision in the defocus in 0-3D range. In the next step, the examination will be repeated using the light sword lens and the ophthalmic pinhole. The last phase of research will be an interview regarding the feelings of the subject, especially in the field of visual comfort. The duration of the experimental session will not exceed 2 hours.

5.8 Nature and degree of all known and anticipated threats (eg undesirable effects) and benefits for the health and life of the subjects as well as cognitive and practical values related to running research

There are no risks associated with conducting the tests, a slight temporary deterioration of contrast vision during the examination (during correction with the light sword lens) is expected without consequences after the test.
The used light sword lens artificially increases the depth of field of vision and should provide visual acuity corresponding to normal vision over the entire normal vision range (0D-3D), therefore can be an effective method of compensating for presbyopia.

5.9 **Proposed evaluation of test results** and **statistical methods to be used for testing**

The percentage of respondents achieving a given visual acuity (VA) for each line will be calculated after performing the test with optometric charts. After deletion of the dependence of these percentages on subsequent VA values, the logistic curve and the designated threshold being the VA value of the line will be adjusted, which on average correctly recognized 50% of respondents as well as the uncertainty associated with the slope of dependence at this point. The final result of the study will be the dependence of

threshold VA values on defocusing vision before and after correcting with a light sword lens and for vision through the eye pinhole.

5.10 In the case of a multicenter study, information on the total extent of the study

Research will be conducted at the Military Institute of Medicine and the Faculty of Physics at the Warsaw University of Technology.

5.11 The form of the "Consent of the patient", and in the case of more complex examinations, the "Patient information" containing a comprehensive and comprehensible description of the study and its possible beneficial and adverse effects on the patient

I agree to participate in an experiment regarding an innovative method of vision correction using optical elements that artificially increase the depth of visual acuity. I am aware that during the research, my task will be to recognize the optotypes on test charts. During the task, I will look through various optical elements placed in ophthalmic frames.

5.1 Formula of:

We declare that we are aware that the approval of research by the Bioethical Commission does not absolve us from medical liability on general terms for health and life entrusted to our care for the sick. At the same time, we declare that we have read and undertake to comply "Rules for the proper conduct of clinical trials of medicinal products in the European Union"

Head of the research project (-)

Members or research team: (-)

(-)

(-)

(-)

(-)

(-)

(-)

..
